# AI-Assisted Brain Tumor MRI Reporting and Treatment-Planning Segmentation: A Retrospective Paired Workflow Evaluation

**DOI:** 10.3390/biomedicines14071595

**Published:** 2026-07-16

**Authors:** Jia-Sheng Hong, Wei-Kai Lee, Jing-Jhong Chen, Yi-Chen Sun, Ying-Yi Hsu, Yung-Fa Lu, Ming-Hsi Sun, Kai-Lin Yang, Chia-Yu Lin, Hsiu-Mei Wu, Shu-Ting Chen, Wan-Yuo Guo, Hung-Chieh Chen, Weir-Chiang You, Yu-Te Wu

**Affiliations:** 1 Institute of Biophotonics, National Yang Ming Chiao Tung University, Taipei 112, Taiwan; eternity.jshong@nycu.edu.tw (J.-S.H.);; 2 Department of Biomedical Science and Technology, National Sun Yat-sen University, Kaohsiung 804, Taiwan; wklee@mail.nsysu.edu.tw; 3 Department of Radiation Oncology, Taichung Veterans General Hospital, Taichung 407, Taiwan; 4 Department of Neurosurgery, Neurological Institute, Taichung Veterans General Hospital, Taichung 407, Taiwan; 5 Department of Radiation Therapy and Oncology, Shin Kong Wu Ho-Su Memorial Hospital, Taipei 111, Taiwan; 6 School of Medicine, Fu Jen Catholic University, New Taipei City 242, Taiwan; 7 Division of Neuroradiology, Department of Radiology, Taichung Veterans General Hospital, Taichung 407, Taiwan; 8 Department of Radiology, Taipei Veterans General Hospital, Taipei 112, Taiwan; 9 School of Medicine, College of Medicine, National Yang Ming Chiao Tung University, Taipei 112, Taiwan; 10 Department of Post-Baccalaureate Medicine, National Chung Hsing University, Taichung 402, Taiwan; 11 Brain Research Center, National Yang Ming Chiao Tung University, Taipei 112, Taiwan; 12 College Medical Device Innovation and Translation Center, National Yang Ming Chiao Tung University, Taipei 112, Taiwan; 13 Center for Smart Health and Medicine, Taipei City Hospital, Taipei 112, Taiwan

**Keywords:** brain tumor, magnetic resonance imaging, artificial intelligence, structured reporting, tumor delineation, radiotherapy planning, AI-assisted workflow, auto-segmentation, neuro-oncology

## Abstract

**Background**: Brain tumor magnetic resonance imaging (MRI) reporting and tumor segmentation for treatment planning are time-consuming and variable. This retrospective fixed-sequence paired workflow study evaluates whether AI assistance is associated with changes in efficiency, consistency, and reproducibility. **Methods**: Thirty MRI cases (10 vestibular schwannomas, 10 meningiomas, 10 brain metastases) were assessed. Two neuroradiologists completed diagnostic reporting with and without AI assistance, and two physicians completed tumor delineation with and without AI-generated preliminary contours after a 3-week washout. **Results**: Reporting time decreased from 42.94 to 27.90 min for Reader A and from 101.04 to 80.47 min for Reader B, corresponding to median paired case-level reductions of 40.39% and 11.51%, respectively; only Reader A reached statistical significance. Sensitivity remained 97.73% and 100.00%, while precision was numerically higher after AI assistance (89.58% to 97.73% and 83.02% to 91.67%). Report-similarity metrics increased across ROUGE-L, BERTScore F1, and Sentence-BERT cosine similarity (all *p* < 0.001). Contouring time decreased from 54.63 to 4.93 min for Reader 1 and from 184.44 to 44.19 min for Reader 2, with median paired reductions of 100.00% and 87.11%. Dice coefficients were numerically higher after AI assistance (0.81 to 0.87 and 0.83 to 0.87). **Conclusions**: AI assistance was associated with shorter task-completion times, higher report-similarity metrics, and numerically higher contour-overlap measures. Prospective validation should determine whether these workflow efficiency gains translate into broader clinical benefit.

## 1. Introduction

Brain tumor management relies heavily on magnetic resonance imaging (MRI) throughout the clinical pathway, from initial detection to lesion characterization, response assessment, and treatment planning. In neuro-oncology, standardized imaging frameworks such as the Response Assessment in Neuro-Oncology (RANO) criteria [1] and the RANO criteria for brain metastases (RANO-BM) [2] emphasize consistent evaluation of lesion size, enhancement pattern, multiplicity, and interval change. In parallel, structured reporting frameworks such as the Brain Tumor Reporting and Data System (BT-RADS) have been introduced to improve the consistency, completeness, and clinical usability of brain tumor MRI reports [3,4]. Together, these developments highlight the need for imaging workflows that are accurate, reproducible, and standardized across readers and clinical settings.

Brain tumor MRI interpretation remains labor-intensive. A prior time-motion analysis of brain MRI interpretation reported that the total interpretation workflow, measured from study opening to report signing, required approximately 10–18 min per case across reader roles, with time distributed among image viewing, report transcription, obtaining clinical data, education, and other related activities [5]. Prolonged reporting turnaround time can affect departmental efficiency and may also influence downstream clinical operations. Assistive artificial intelligence (AI), including structured reporting tools and large language model (LLM)-based support systems, has therefore attracted interest as a means of improving reporting efficiency, standardization, and quality [6,7]. Although recent studies have reported favorable performance for AI-assisted reporting paradigms, their value in disease-specific clinical brain tumor workflows remains to be validated.

Artificial intelligence (AI) includes conventional machine learning models and deep-learning methods that learn imaging representations directly from data. These approaches differ in data requirements, interpretability, and validation needs. Recent systematic reviews in neurosurgical, neuroendocrine, and neuroimaging applications highlight rapid growth of clinical AI while emphasizing heterogeneity and the need for external validation [8,9,10].

A similar challenge exists on the treatment side. Tumor delineation for surgery and radiotherapy planning is time-consuming and subject to interobserver variability, particularly when lesions are multiple, irregular, or adjacent to critical neuroanatomical structures [11]. Recent reviews and clinical evaluations have shown that AI-based auto-segmentation can reduce contouring burden and improve reproducibility when used under physician supervision [12]. In brain tumor imaging, meta-analytic evidence suggests that deep learning models have demonstrated performance in detection and segmentation across brain metastases, meningiomas, gliomas, and vestibular schwannomas, supporting their use as assistive tools in treatment planning rather than as fully autonomous systems [13,14,15,16].

However, most prior studies have evaluated AI assistance for diagnostic reporting and contouring separately. In brain tumor care, these processes are clinically connected: diagnostic MRI interpretation informs follow-up decisions, multidisciplinary treatment strategy, target delineation, and treatment delivery. AI-assisted tools may be useful not only for isolated algorithmic tasks, but also for creating shared lesion-level information that can be carried from diagnostic reporting into treatment planning. When lesions are preliminarily marked by AI during image review, physicians may verify report content, communicate lesion location and extent, and transfer relevant imaging information to downstream contouring workflows.

**Figure 1:**
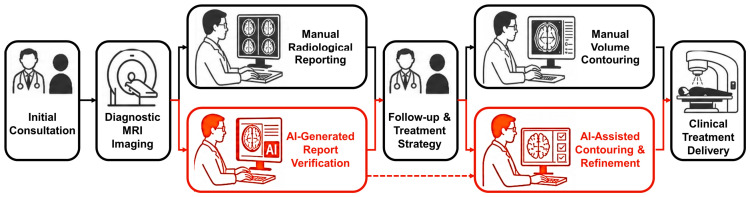
AI-assisted neuro-oncology imaging workflow.

For physician-facing AI tools, workflow efficiency is a key clinical consideration. Even if an AI model demonstrates acceptable standalone diagnostic or segmentation performance, its clinical utility may be limited if its use increases reader workload, prolongs reporting or contouring time, or disrupts routine practice. From this perspective, time efficiency provides an appropriate primary endpoint for workflow evaluation: an AI-assisted workflow should reduce, or at least not increase, the physician burden required to complete clinical tasks. Measures of lesion detection, report consistency, segmentation overlap, and volumetric agreement were therefore considered complementary outcomes to determine whether any time savings were achieved without compromising output quality. This rationale led to the present study, which evaluates whether AI assistance is associated with changes in workflow efficiency, consistency, and reproducibility in two connected use scenarios: brain tumor MRI report preparation and treatment-planning tumor delineation, using controlled retrospective paired workflow testing (Figure 1).

## 2. Materials and Methods

### 2.1. Study Design

This retrospective controlled study evaluated physician workflows with and without AI assistance in two linked clinical tasks: diagnostic MRI reporting and treatment-planning tumor delineation. The evaluation consisted of two stages: first, the establishment of a consensus reference standard from 30 retrospective MRI cases by an independent three-physician panel external to the study reader group; second, task-specific reader evaluations using the same cases. The reference-standard physicians did not participate in the subsequent diagnostic reporting or treatment-planning delineation reader-evaluation sessions. In both tasks, the AI-assisted session was performed first, followed by a 3-week washout period and then the physician-only session. Although the same 30 cases were used across sessions, case identifiers were de-identified and case presentation order was randomized separately for each physician and each session to reduce case recognition, sequence effects, and recall bias. The workflow condition order was fixed, with AI-assisted sessions performed before physician-only sessions. This sequence was selected under the assumption that any residual case familiarity after the washout would be more likely to benefit the later physician-only session rather than the earlier AI-assisted session, thereby reducing the likelihood of overestimating the apparent effect of AI assistance. However, the direction and magnitude of order effects cannot be determined from this design; therefore, the study should be interpreted as a fixed-sequence paired comparison rather than a randomized or counterbalanced design. Recall, learning, case familiarity, fatigue, or workflow adaptation may still have influenced the results despite the washout.

For diagnostic reporting, two readers generated brain tumor MRI reports under both AI-assisted and physician-only conditions. In the AI-assisted condition, MRI-based lesion localization and structured lesion-level information derived from the AI output were available for physician review and report preparation; in the physician-only condition, readers interpreted the same cases without AI support. Report generation time was measured from the start of image review to completion of the finalized report, thereby including image review, interpretation, and report generation. These measurements did not include worklist queue time, retrieval of clinical history outside the study materials, comparison with prior examinations outside the supplied study images, report-signing delay, or clinician notification.

**Figure 2:**
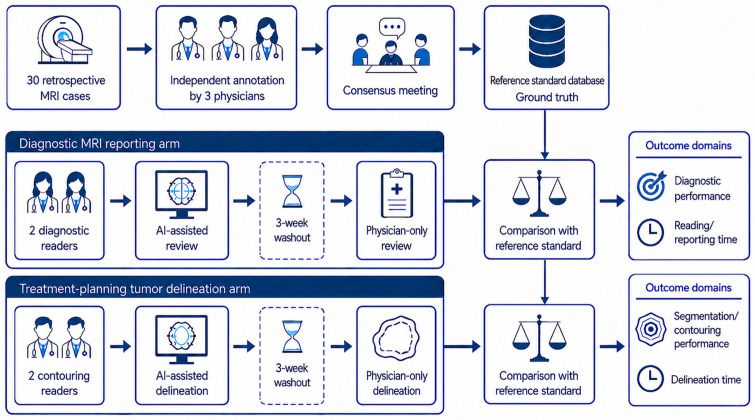
Retrospective paired workflow evaluation design.

For treatment-planning tumor delineation, two contouring readers generated final lesion contours under both AI-assisted and physician-only conditions. In the AI-assisted condition, readers reviewed and edited the AI system-generated preliminary tumor contours as needed before finalization; in the physician-only condition, readers manually delineated the same cases without AI support. Delineation time was recorded separately for each individual lesion. For cases with multiple lesions, the active drawing, editing, or contour-confirmation time for each lesion was measured independently rather than only as a single total case time. The recorded time reflected only active contouring or editing activity; image browsing, case review, and software operation time outside manual contouring or editing were excluded. These task-specific timing definitions reflect the different workflow endpoints of the two experiments: the reporting experiment evaluated the time required to review images and produce a finalized report, whereas the contouring experiment evaluated the active physician delineation or editing time required to generate treatment-planning contours. The overall study design and workflow are summarized in Figure 2.

### 2.2. Sample Size Rationale and Power Consideration

This study was designed as a retrospective paired workflow-efficiency evaluation rather than a comprehensive validation study of lesion-level diagnostic or segmentation performance. Because each case was evaluated under both physician-only and AI-assisted conditions, the primary analysis was planned as a paired, within-case comparison. The primary workflow-efficiency variable was defined as the case-level relative workflow–time reduction:

$$R_{i}=\frac{T_{PO,i}-T_{AI,i}}{T_{PO,i}}$$

where *R_i_* is the relative workflow–time reduction for case *i*, *T_PO,i_* is the time required under the physician-only condition, and *T_AI,i_* is the time required under the AI-assisted condition.

Because reliable prior estimates of the standard deviation of paired relative workflow–time reduction were not available for this specific clinical setting, the sample size rationale was based on a standardized paired-effect framework and estimation precision rather than on a fixed absolute time-reduction threshold. With 30 paired cases, the study provides approximately 80% power to detect a medium standardized paired effect size (*d_z_* = 0.53) using a two-sided paired-comparison framework at alpha = 0.05 [17]. In this context, *d_z_* represents the mean paired workflow–time difference divided by the standard deviation of the paired differences; it is a standardized measure of paired workflow change rather than a percentage time reduction. In addition, 30 paired cases allow the mean paired relative workflow–time reduction to be estimated with a 95% confidence interval half-width of approximately 0.37 standard deviations, providing a precision-based rationale for estimating the workflow–time effect and its variability. The same sample size also allowed balanced representation of the three target tumor categories. Therefore, 30 retrospective paired cases were included, with 10 cases each of vestibular schwannoma, meningioma, and brain metastasis. Lesion-level sensitivity, precision, report consistency, Dice coefficient, and volumetric agreement were treated as secondary descriptive performance measures and were not used as the basis for sample size determination.

### 2.3. Study Population and Image Acquisition

This retrospective study was approved by the Institutional Review Board (IRB; protocol code SE24344C; approval date: 23 July 2024), and informed consent was waived because only de-identified retrospective imaging data were analyzed. Cases were retrospectively selected to provide 10 cases in each target tumor category and required diagnostic-quality contrast-enhanced T1-weighted and T2-weighted imaging; the cohort was not intended to represent a consecutive or population-based sample. This study included MRI data from a total of 30 clinical cases, comprising 10 vestibular schwannomas, 10 meningiomas, and 10 brain metastases. The patient cohort consisted of 21 females and 9 males, with a mean age of 60.2 ± 13.1 years (range: 32–84 years).

All imaging data were acquired using either 1.5 Tesla (1.5 T; *n* = 18, 60.0%) or 3.0 Tesla (3.0 T; *n* = 12, 40.0%) MRI systems to reflect real-world clinical heterogeneity. Examinations were performed on multi-vendor platforms, including GE Healthcare scanners (Signa HDxt and Optima MR450w; *n* = 19), Siemens Healthineers scanners (MAGNETOM Verio and Lumina; *n* = 6), and Philips Healthcare scanners (Ingenia Elition X; *n* = 5).

The standard imaging protocol for all cases included multi-contrast sequences, specifically incorporating both contrast-enhanced T1-weighted imaging (CE-T1WI) and T2-weighted imaging (T2WI). Detailed acquisition parameters varied according to institutional protocols and scanner models. Slice thickness ranged from 0.9 mm to 3.0 mm, with 3.0 mm being the most prevalent value (66.7%).

### 2.4. Reference Standard and Physician Participants

A reference standard dataset was established before formal workflow evaluation by an independent three-physician panel external to the study reader group. The panel physicians did not participate in the diagnostic reporting or treatment-planning delineation experiments. Three board-certified radiology/neuroradiology attending physicians, each with more than 10 years of clinical experience, independently reviewed and annotated the 30 included cases. After independent annotation, lesion candidates were compared across the three physicians. Lesions annotated by two or more physicians were included in the reference standard, and the union of their annotated regions was used as the initial ground-truth contour. Lesions annotated by only one physician were flagged for consensus review. During the consensus meeting, the three physicians jointly reviewed these single-reader lesion candidates, determined whether each lesion should be included in the reference standard, and confirmed or adjusted the final contour boundary when inclusion was agreed upon. The final reference standard database, therefore, incorporated both multi-reader lesion agreement and consensus adjudication for discordant or single-reader findings. This database served as the ground truth against which physician-only, AI-assisted physician, and AI-generated preliminary outputs were compared. The union-based contour strategy was used to reduce exclusion of potential lesion extent, but it may influence precision and overlap metrics compared with majority-vote or probabilistic fusion methods.

Four physicians participated in the study. The diagnostic reporting task was performed by two neuroradiologists with 25 and 10 years of clinical experience in brain MRI interpretation, respectively. The treatment-planning segmentation task was performed by one neurosurgeon with 35 years of experience and one radiation oncologist with 10 years of experience in neuro-oncology treatment planning and contouring. All readers evaluated both AI-assisted and non-AI-assisted workflows within their respective tasks. For each evaluation arm, readers were selected to capture variation in clinical experience, allowing assessment of workflow effects across physicians with different levels of task-specific expertise. Because only two readers performed each task, reader-level results were interpreted descriptively.

### 2.5. AI System

**Figure 3:**
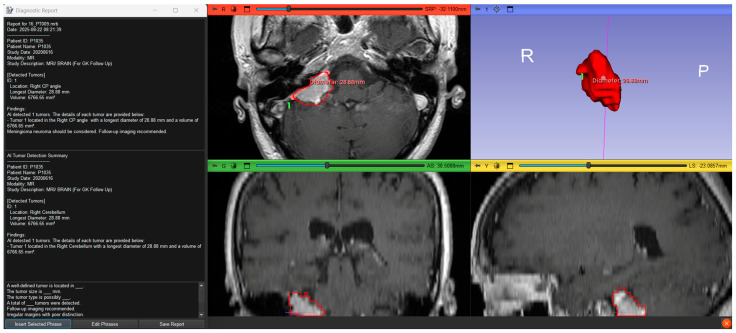
Representative AI assistance interface in 3D Slicer. The left side shows structured lesion-level information used during physician report preparation, and the right side shows MRI-based lesion localization and contouring support.

DeepBT Detector-Plus v1.0.0 (AItewan BioMedical Technology Inc., Taipei, Taiwan) is an AI-assisted software-as-a-medical-device (SaMD) product for brain tumor image analysis in radiotherapy-related treatment-planning workflows. Regulatory identifiers include the Taiwan Food and Drug Administration (TFDA) medical device license (MOHW-MD-No. 008460) and the U.S. Food and Drug Administration (FDA) 510(k) clearance (K252190). In this study, the AI assistance primarily provided MRI-based lesion localization and preliminary tumor contours for physician review. For the diagnostic-reporting experiment, the study interface also displayed structured lesion-level information derived from the AI output, including lesion location and size-related measurements, to support physician review and report preparation (Figure 3). These data were presented as structured information rather than as an automatically finalized radiology report; readers reviewed the MRI images and finalized all report content themselves.

To standardize the experimental environment and minimize interface-related variability, reference-standard annotation, diagnostic reporting, and tumor delineation experiments were conducted using the same reader interface in 3D Slicer (version 5.8.0). This controlled environment was used to review MRI examinations, reference-standard annotations, AI-generated outputs, and physician-generated outputs, and to record task-timing procedures across reference-standard generation, AI-assisted sessions, and physician-only sessions.

### 2.6. Outcome Measures

#### 2.6.1. Diagnostic Reporting Outcomes

The diagnostic reporting evaluation included reporting efficiency, lesion-level diagnostic performance, and report consistency. Reporting efficiency was assessed using the total report generation time across the 30 cases for each reader. Diagnostic performance was assessed using lesion-level sensitivity and precision against the reference standard. Report consistency was assessed using Recall-Oriented Understudy for Gisting Evaluation-Longest Common Subsequence (ROUGE-L), BERTScore, and Sentence-BERT (SBERT) cosine similarity to quantify structural and semantic similarity between reports generated by different readers. ROUGE-L was used as a lexical and structural overlap metric based on the longest common subsequence between reports [18]. BERTScore F1 was used to quantify token-level semantic similarity using contextual BERT embeddings [19]. SBERT cosine similarity was used to compare sentence-level embeddings as a global semantic similarity measure between reports [20].

#### 2.6.2. Treatment-Planning Outcomes

The treatment-planning evaluation included contouring efficiency, volumetric agreement, lesion-level contouring performance, and segmentation overlap. Contouring efficiency was assessed using per-lesion delineation or contour-editing time across the 30 cases for each reader, with lesion-level times summed within each case for paired case-level workflow–time comparisons. Volumetric agreement was assessed by comparing tumor volumes among AI contours, manual physician contours, and AI-assisted physician contours. Lesion-level contouring performance was assessed using sensitivity and precision, and segmentation overlap was evaluated using the Dice coefficient.

### 2.7. Statistical Analysis and Computational Tools

Descriptive statistics were used to summarize workflow efficiency, diagnostic performance, contouring performance, and report consistency. Continuous variables were summarized as means with standard deviations and median with interquartile range (IQR), as appropriate.

For workflow–time analyses, case-level paired observations were used. Report generation time was analyzed at the case level. For tumor delineation, the raw time unit was the individual lesion; when a case contained more than one lesion, all lesion-level edit times within that case were summed to obtain case-level contouring time before paired comparison. Paired workflow–time comparisons between AI-assisted and physician-only conditions were performed using two-sided Wilcoxon signed-rank tests. Case-level relative workflow–time reductions were calculated for each paired case and summarized using median and IQR.

Cross-reader report-similarity metrics (ROUGE-L, BERTScore F1, and SBERT cosine similarity) were compared between physician-only and AI-assisted reports using paired two-sided Wilcoxon signed-rank tests across cases. Lesion-level sensitivity, precision, and Dice coefficient were summarized descriptively without formal hypothesis testing or confidence-interval-based inference. These metrics were considered secondary descriptive performance measures. Tumor volume differences across standalone AI contours, manual physician contours, and AI-assisted physician contours were assessed using the Kruskal–Wallis test.

Linear least-squares regression analysis was performed to examine the relationship between tumor volume and manual contouring or editing time, and regression results were reported using fitted equations and coefficients of determination (*R*^2^). A *p*-value < 0.05 was considered statistically significant. No formal adjustment for multiple comparisons was applied; therefore, *p*-values for non-primary analyses should be interpreted cautiously.

All statistical analyses were performed using Python (version 3.12.12). Data handling and numerical calculations were performed using pandas (version 2.3.3) and NumPy (version 2.3.5). Statistical testing and regression analyses were performed using SciPy (version 1.16.3) and statsmodels (version 0.14.6). Report-similarity metrics were calculated using rouge-score (version 0.1.2) for ROUGE-L, bert-score (version 0.3.13) for BERTScore, and sentence-transformers (version 5.1.2) with scikit-learn (version 1.7.2) for Sentence-BERT cosine similarity. Figures were generated using Matplotlib (version 3.10.8) and seaborn (version 0.13.2).

### 2.8. Preparation of Schematic Figures and AI-Assisted Manuscript Support

Figure 1 and Figure 2 were prepared as schematic workflow illustrations during manuscript preparation. Initial figure concepts and layouts were generated using ChatGPT (OpenAI) from author-provided descriptions of the clinical imaging workflow and the retrospective paired evaluation design. The authors reviewed, edited, and finalized the schematic content to ensure consistency with the study design and manuscript text. ChatGPT was not used to generate, modify, analyze, or interpret patient-level data, clinical images, statistical results, or study conclusions.

## 3. Results

### 3.1. Diagnostic Reporting Performance

**Figure 4:**
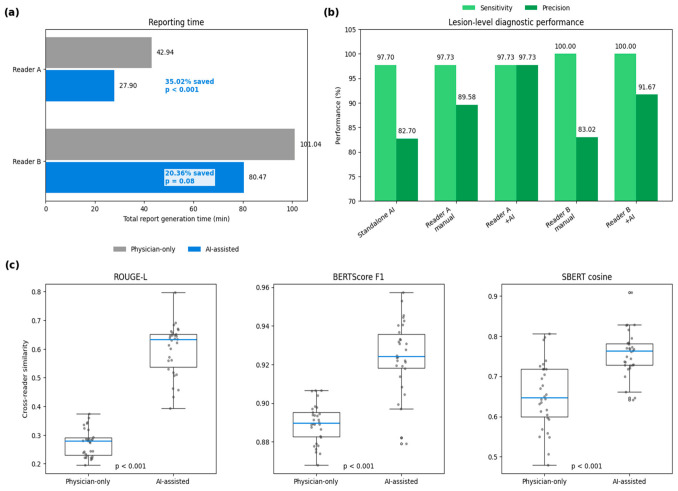
Diagnostic MRI reporting outcomes. (**a**) Total report generation time under physician-only and AI-assisted conditions. (**b**) Lesion-level sensitivity and precision. (**c**) Cross-reader report consistency assessed by ROUGE-L, BERTScore F1, and Sentence-BERT cosine similarity.

AI assistance reduced the total time to generate reports for both neuroradiologists, although case-level variability differed between readers. For the neuroradiologist with 25 years of experience, total reporting time across 30 cases decreased from 42.94 min without AI assistance to 27.90 min with AI assistance, corresponding to a 35.02% total-time reduction. On a paired case-level basis, mean reporting time decreased from 1.43 ± 0.59 to 0.93 ± 0.50 min per case, and median reporting time decreased from 1.29 min (IQR, 1.04–1.54) to 0.78 min (IQR, 0.62–1.07). The median paired relative reporting-time reduction was 40.39% (IQR, 19.64–49.89%; two-sided Wilcoxon signed-rank test, *p* < 0.001). For the neuroradiologist with 10 years of experience, total reporting time decreased from 101.04 to 80.47 min, corresponding to a 20.36% total-time reduction. At the case level, mean reporting time decreased from 3.37 ± 2.12 to 2.68 ± 2.00 min per case, and median reporting time decreased from 2.79 min (IQR, 1.90–3.93) to 2.00 min (IQR, 1.58–3.16). The median paired relative reporting-time reduction was 11.51% (IQR, −7.80 to 34.68%; two-sided Wilcoxon signed-rank test, *p* = 0.08), indicating numerical time savings with greater case-level variability. Sensitivity remained 97.73% and 100.00% for the two readers, respectively, whereas precision was numerically higher after AI assistance, increasing from 89.58% to 97.73% and from 83.02% to 91.67%. Because sensitivity and precision were secondary descriptive performance measures, these findings should be interpreted as supportive rather than confirmatory. The AI tool alone showed sensitivity of 97.70% and precision of 82.70%, but the primary analysis focused on physician workflow performance with and without AI assistance. The main observed diagnostic benefits were shorter reporting time and higher report-similarity metrics, with descriptively fewer false-positive interpretations. Reporting-time results are shown in Figure 4a, and lesion-level diagnostic sensitivity and precision are shown in Figure 4b.

### 3.2. Inter-Reader Report Consistency

Cross-reader report consistency was evaluated by comparing the two diagnostic readers’ reports for the same case under physician-only and AI-assisted conditions. As shown in Figure 4c, AI-assisted reporting produced higher cross-reader similarity than physician-only reporting across all three natural language processing metrics: ROUGE-L, BERTScore F1, and SBERT cosine similarity (all *p* < 0.001). These findings indicate that AI support was associated with greater structural and semantic standardization of brain tumor MRI reports between readers. These metrics evaluate textual similarity and should not be interpreted as direct measures of clinical report quality.

### 3.3. Tumor Delineation Performance

**Figure 5:**
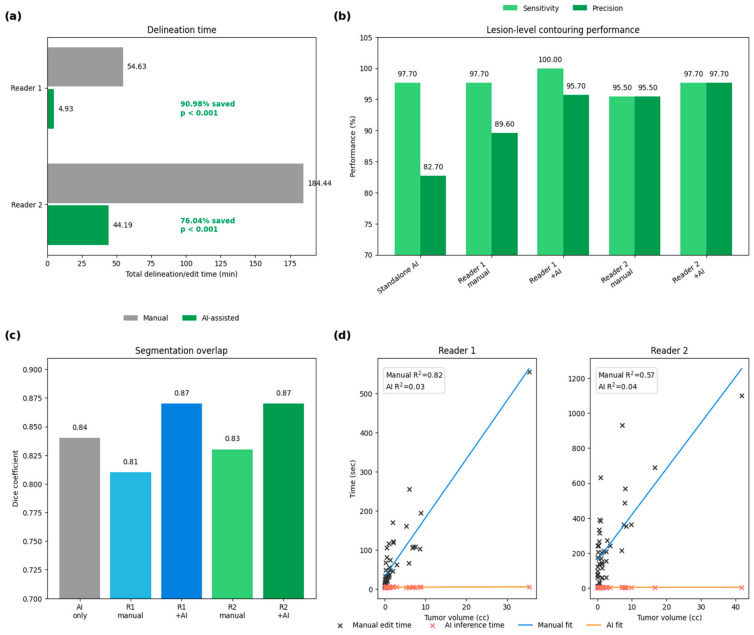
Treatment-planning tumor delineation outcomes. (**a**) Total delineation/edit time under manual and AI-assisted conditions. (**b**) Lesion-level sensitivity and precision. (**c**) Dice coefficient. (**d**) Tumor volume versus manual edit time and AI inference time for the two contouring readers.

In the treatment-planning evaluation, AI assistance reduced total contouring time for both participating physicians. For the neurosurgeon with 35 years of experience, total contouring time across 30 cases decreased from 54.63 min with manual contouring to 4.93 min with AI-assisted contour editing, corresponding to a 90.98% total-time reduction. On a paired case-level basis, mean contouring time decreased from 1.82 ± 1.65 to 0.16 ± 0.74 min per case, and median contouring time decreased from 1.52 min (IQR, 0.94–1.96) to 0.00 min (IQR, 0.00–0.00). The median paired relative contouring-time reduction was 100.00% (IQR, 100.00–100.00%; two-sided Wilcoxon signed-rank test, *p* < 0.001). For the radiation oncologist with 10 years of experience, total contouring time decreased from 184.44 to 44.19 min, corresponding to a 76.04% total-time reduction. At the case level, mean contouring time decreased from 6.15 ± 5.14 to 1.47 ± 2.10 min per case, and median contouring time decreased from 4.06 min (IQR, 2.45–7.92) to 0.57 min (IQR, 0.29–1.74). The median paired relative contouring-time reduction was 87.11% (IQR, 67.80–90.71%; two-sided Wilcoxon signed-rank test, *p* < 0.001). For the first contouring reader, sensitivity, precision, and Dice coefficient were numerically higher after AI assistance (97.70% to 100.00%, 89.60% to 95.70%, and 0.81 to 0.87, respectively). For the second contouring reader, sensitivity, precision, and Dice coefficient were also numerically higher after AI assistance (95.50% to 97.70%, 95.50% to 97.70%, and 0.83 to 0.87, respectively). The standalone AI contour output showed sensitivity of 97.70%, precision of 82.70%, and a Dice coefficient of 0.84; however, the primary treatment-planning analysis focused on physician-finalized contours generated with and without AI assistance. Because these metrics were secondary descriptive performance measures, the findings should be interpreted as reduced active contouring burden with supportive contouring metrics rather than confirmatory evidence of improved segmentation performance. Contouring-time results are shown in Figure 5a, lesion-level sensitivity and precision are shown in Figure 5b, and Dice coefficients are shown in Figure 5c.

### 3.4. Volume Agreement

Comparison of tumor volumes among standalone AI contours, manual physician contours, and AI-assisted physician contours showed no significant intergroup difference (Kruskal–Wallis test, *p* = 0.705). This finding suggests that the AI system did not introduce a systematic volumetric difference relative to expert-generated contours.

### 3.5. Relationship Between Tumor Size and Contouring Time

Manual contouring time increased with tumor volume, indicating greater physician burden in larger lesions. On lesion-level regression analysis, manual edit time increased with tumor volume for both contouring readers: Reader 1 showed a fitted relationship of *y* = 15.00 *x* + 31.88 s (*R*^2^ = 0.82), and Reader 2 showed a fitted relationship of *y* = 26.28 *x* + 156.95 s (*R*^2^ = 0.57), where x represents tumor volume in cubic centimeters. In contrast, AI inference time showed minimal dependence on tumor volume, with fitted relationships of *y* = 0.02 *x* + 4.42 s (*R*^2^ = 0.03) for Reader 1 cases and *y* = 0.02 *x* + 4.43 s (*R*^2^ = 0.04) for Reader 2 cases. These findings suggest that AI support may mitigate the volume-dependent workload associated with manual delineation, particularly for larger or more complex lesions. The volume–time relationship is shown in Figure 5d.

## 4. Discussion

In this study, we evaluated the workflow effects of AI assistance across two connected tasks in brain tumor care: MRI reporting and tumor delineation for treatment planning. AI assistance was associated with shorter task-completion times in both settings, higher report-similarity metrics, and numerically higher contour-overlap measures. These findings suggest that the value of AI in this setting should be assessed not only by standalone algorithmic performance but also by its ability to support physician-supervised, standardized clinical workflows. This interpretation is consistent with prior work emphasizing structured reporting in neuro-oncology, LLM-supported radiology reporting, and AI-based segmentation as tools for workflow standardization rather than replacements for physician judgment [3,4,5,6,7,12].

The clinical contribution of AI differed between the diagnostic and treatment-planning settings. In the diagnostic workflow, the main observed benefit was shorter reporting time and higher inter-reader report-similarity metrics rather than increased sensitivity. This pattern is relevant because experienced physicians may already identify most clinically relevant lesions, whereas variability more commonly arises in report structure, description, and emphasis. By providing structured lesion-level information and preliminary annotations, the AI-assisted workflow appeared to reduce inter-reader variation and promote more standardized reporting. However, higher similarity does not necessarily indicate better clinical report quality and may partly reflect anchoring to AI-derived structured information. This finding is aligned with the rationale of BT-RADS and structured reporting frameworks, which aim to improve clarity, completeness, and actionability of brain tumor reports [3,4]. Broader structured-reporting evidence similarly suggests that reporting support may add value by improving completeness, consistency, and usability rather than by replacing expert interpretation [21,22].

In the treatment-planning task, AI assistance reduced contouring burden while producing numerically higher overlap metrics relative to the reference standard. Manual delineation is labor-intensive and depends on lesion complexity, imaging quality, and physician experience; interobserver variability in brain tumor gross tumor volume delineation has been documented even when MRI information is available [11]. In this study, AI-generated preliminary contours shifted physician work from full manual segmentation toward review-and-edit behavior, which likely explains the observed reduction in annotation time. For Reader 1, the median AI-assisted edit time was 0.00 min, indicating that in many cases the physician accepted the AI-generated contour after review without additional manual modification; these zero-time observations therefore reflect contour confirmation rather than missing measurements. These findings are consistent with recent deep learning applications in MRI-guided radiotherapy and brain tumor segmentation, where AI supports segmentation while preserving physician oversight [12,13,14,15,16,23,24,25,26,27]. Clinical implementation studies of auto-segmentation also emphasize that physician review, local validation, editing burden, and clinical acceptability should be evaluated alongside geometric metrics such as the Dice coefficient [28,29,30]. Nevertheless, geometric metrics do not fully establish clinical acceptability, and AI contours require physician review, particularly near critical neurovascular structures.

The volume–time regression provides an additional interpretation of manual contouring burden. For manual delineation, each additional cubic centimeter of tumor volume was associated with approximately 15.00 s and 26.28 s longer active edit time for Readers 1 and 2, respectively. The intercepts should not be interpreted as the expected time for a true zero-volume lesion, because zero volume is outside the clinically meaningful range of this task and the intercept is an extrapolated parameter of the fitted linear model. In this analysis, the intercept more appropriately reflects reader-specific baseline active-editing time not explained by tumor volume alone, such as contour initiation, minimum editing steps, slice-by-slice boundary refinement, editing granularity, and lesion-complexity factors not captured by volume. The larger intercept and steeper slope observed for Reader 2 suggest both a higher baseline active-editing burden and a stronger volume-dependent increase in manual contouring time.

The linked reporting-and-contouring workflow may also support more quantitative longitudinal assessment and interdisciplinary communication. In routine clinical practice, treatment response assessment is often constrained by workload and may rely on qualitative review or semi-quantitative measurements such as the longest lesion diameter. AI-assisted contouring can provide volumetric information that may make serial comparison more reproducible. In a longitudinal vestibular schwannoma radiosurgery study, Lee et al. used AI-derived volumetric analysis to track post-treatment tumor changes over serial MRI follow-ups, illustrating how automated volume information can complement conventional response assessment [31]. In the present study, physician-reviewed AI contours may provide quantitative lesion-volume information for both treatment planning and future follow-up comparison, while lesion-level information generated during reporting may help maintain continuity across diagnostic and treatment teams [1,2,3,4].

These workflow effects may also be relevant to radiology turnaround time, although turnaround time is influenced by more than the time needed to compose a report. In routine practice, overall turnaround time can be affected by case arrival and worklist position, prioritization or triage, the interval before a physician opens the examination, image interpretation, report generation, finalization, notification of actionable findings, and clinician review of the report [32,33]. In this context, AI assistance could affect different components of the pathway: automated triage or notification could help identify brain tumor cases requiring earlier physician attention, whereas AI-derived structured lesion-level information may support report preparation once the case is opened. These possibilities should be examined in future studies that integrate AI-assisted reporting with worklist prioritization and notification systems.

Taken together, prior evidence indicates that AI assistance in radiology and radiotherapy is most informative when evaluated as part of a physician-supervised workflow rather than as an isolated algorithmic output. Current guidance for clinical AI evaluation emphasizes human–AI interaction, the intended setting of use, workflow integration, error analysis, external validation, and outcome-oriented assessment [34,35,36,37,38,39,40]. The present work extends this perspective by measuring reporting time, report consistency, contouring time, lesion-level detection performance, volumetric agreement, and Dice overlap in the same paired evaluation framework. Prior reader-assistance, reporting, and auto-contouring studies have reported reductions in reading, reporting, segmentation, or contouring workload, but the magnitude of benefit varies across task definitions, measurement methods, and clinical settings [41,42,43]. By evaluating two connected clinical nodes, this study highlights the potential role of AI assistance in linking diagnostic report preparation with treatment-planning tumor delineation.

This study has several limitations. First, the cohort was small, retrospective, and limited to vestibular schwannoma, meningioma, and brain metastasis; therefore, the findings should not be generalized to gliomas or other tumor types without further validation. The sample size was planned for a paired workflow–time evaluation with power and precision considerations, and the cohort was balanced across the three target tumor categories; nevertheless, the study was not intended to provide comprehensive validation of diagnostic accuracy, segmentation performance, or downstream clinical outcomes. Second, the workflow order was fixed, with AI-assisted sessions performed before physician-only sessions; recall, learning, fatigue, and workflow-adaptation effects cannot be excluded. Third, only two readers performed each task, so reader-level results may reflect individual practice patterns. The inclusion of one more experienced and one less experienced reader in each task provides preliminary observations across experience levels, but the study was not powered to formally test reader–experience interactions. Fourth, reporting and contouring times were controlled task-completion times rather than full clinical turnaround or treatment-planning workflow times. The diagnostic reporting experiment measured report preparation for a single MRI examination under a controlled study setting. In routine clinical practice, physicians often review prior imaging examinations, compare interval changes, consult clinical history, and incorporate information from the medical record before finalizing a brain tumor MRI report. Therefore, the absolute reporting times observed here should not be interpreted as the full clinical reporting time required in routine practice. This study focused on proximal workflow outcomes and did not directly measure clinical turnaround time for brain tumor cases, including time from examination completion to report initiation, final report availability, clinician notification, treatment-planning initiation, or treatment delivery. Fifth, sensitivity, precision, Dice coefficient, and volume agreement were secondary descriptive performance measures without formal confidence intervals or multiplicity adjustment. Sixth, the union-based reference contours, report-similarity metrics, and geometric contour metrics do not fully establish clinical report quality or contour acceptability, and AI outputs may anchor reader decisions. Finally, several authors are affiliated with the developer of the evaluated AI software; independent external validation and prospective multicenter testing are needed.

## 5. Conclusions

This retrospective paired workflow evaluation suggests that AI-assisted workflows are associated with shorter task-completion times, higher report-similarity metrics, and numerically higher contour-overlap measures in brain tumor MRI reporting and treatment-planning delineation. These findings should be interpreted as preliminary workflow evidence from a small fixed-sequence retrospective cohort rather than as validation of standalone AI diagnostic or segmentation performance.

Future prospective multicenter studies with randomized or counterbalanced workflow order, broader representation of tumor types including gliomas, independent external validation, and clinically relevant endpoints such as turnaround time and treatment-planning impact will help determine the extent to which these observed workflow efficiency gains translate into broader clinical benefit.

## Data Availability

The datasets analyzed during the current study are not publicly available because access to the de-identified clinical imaging data is restricted by Institutional Review Board approval and institutional data-governance requirements.
